# Cost-effectiveness of dental caries prevention strategies in South African schools

**DOI:** 10.1186/s12903-023-03474-1

**Published:** 2023-10-28

**Authors:** Micheal Kofi Boachie, Mpho Molete, Karen Hofman, Evelyn Thsehla

**Affiliations:** 1 Discipline of Public Health Medicine, School of Nursing and Public Health, College of Health Sciences, University of KwaZulu-Natal, 4041 Durban, South Africa; 2 SAMRC/Centre for Health Economics and Decision Science - PRICELESS SA, School of Public Health, Faculty of Health Sciences, University of the Witwatersrand, 2193 Parktown, Johannesburg South Africa; 3 Department of Oral Biological Sciences, School of Oral Health Sciences, Faculty of Health Sciences, University of the Witwatersrand, 2193 Parktown, Johannesburg South Africa

**Keywords:** Dental caries, Cost-effectiveness, School health promotion sugar-reduction, School learners, Oral health, South Africa

## Abstract

**Background:**

In South Africa, an estimated 85% of the population relies on the public sector for oral health services. With poor infrastructure and inadequate personnel, over 80% of children with dental caries remain untreated. To reduce this burden of disease, one key goal is to promote good oral health and address oral diseases through prevention, screening, and treatment among children. While all policies have been proven to be effective in the control and prevention of dental caries, it is unclear which of those strategies provide value for money. This study evaluated five caries preventative strategies in terms of the cost and benefits among South African school children.

**Methods:**

The study uses a hypothetical South African population of school aged learners aged 5–15. The context and insights of the strategies utilized at the schools were informed by data from both grey and published literature. Using Markov modeling techniques, we conducted a cost-effectiveness analysis of Acidulated Phosphate Fluoride (APF) application, atraumatic restorative treatment (ART), sugar-reduction and fissure sealants. Markov model was used to depict the movement of a hypothetical patient cohort between different health states over time. We assessed both health outcomes and costs of various interventions. The health outcome metric was measured as the number of Decayed, Missing, Filled Tooth (DMFT). The net monetary benefit was then used to determine which intervention was most cost-effective.

**Results:**

The results showed that school-based caries prevention strategies are cost-effective compared to the status quo of doing nothing. The average cost per learner over the 10-year period ranged from ZAR4380 to approx. ZAR7300 for the interventions considered. The total costs (including screening) associated with the interventions and health outcome (DMFT averted) were: sugar reduction (ZAR91,380, DFMT: 63,762), APF-Gel (ZAR54 million, DMFT: 42,010), tooth brushing (ZAR72.8 million, DMFT: 74,018), fissure sealant (ZAR44.63 million, DMFT: 100,024), and ART (ZAR45 million, DMFT: 144,035). The net monetary benefits achieved for APF-Gel, sugar reduction, tooth brushing, fissure sealant and ART programs were ZAR1.56, ZAR2.45, ZAR2.78, ZAR3.81, and ZAR5.55 billion, respectively.

**Conclusion:**

Based on the net monetary benefit, ART, fissure sealant and sugar-reduction appear to be the most cost-effective strategies for preventing caries in South Africa. In a resource-scarce setting such as South Africa, where there is no fluoridation of drinking water, this analysis can inform decisions about service packages for oral health.

**Supplementary Information:**

The online version contains supplementary material available at 10.1186/s12903-023-03474-1.

## Background

One of the neglected public health issues globally is dental caries and periodontal diseases, partly because direct mortality from oral diseases is rare [1]. Though most oral disorders are preventable, in most low- and middle-income countries (LMICs) there is significant morbidity if left untreated [1]. This contributes to the high level of disability-adjusted life years (DALYs) from oral health disorders. Globally, untreated caries in primary dentition increased by 1.3% between 1990 and 2017; untreated permanent dentition caries increased by 36% during the same period [1].

Caries experience is age dependent, [2–4] and its high prevalence of caries together with other oral health conditions has been linked to poor oral hygiene from high levels of sugar consumption and poor oral care habits [5–7]. It has been established that tooth decay in children and adults is mainly caused by sugar [7]. In fact, both the quantity and frequency of sugar intake have been linked to caries, [8, 9] with children who consume large quantities of sugar have about 50% risk of developing caries [10]. A recent study of 5-15-year-olds in India showed that children who consumed sugar frequently had 32% likelihood of developing dental caries [11]. Among adults, studies have shown that consuming sugar sweetened beverages (SSBs) three times daily contributed 33% to caries increment, regardless of the fluoride exposure [12, 13]. In 2010, 26.3% of the total oral disease burden was due to added sugar consumption [14]. This is because sugary foods mixes with the bacteria in the mouth, causing bacterial plaque which leads to dental caries and gingivitis or gum disease. When the two conditions are left unattended, they can lead to tooth loss and chewing dysfunction. [15].

Poor oral health has also been identified as a risk factor for other non-communicable diseases (NCDs) such as diabetes and obesity [16]. This means that oral diseases share risk factors with NCDs. Therefore prevention of shared risks such as prohibition of sugary drinks and snacks among children has the potential of not only addressing dental caries but common NCDs as well [17].

**Fig. 1:**
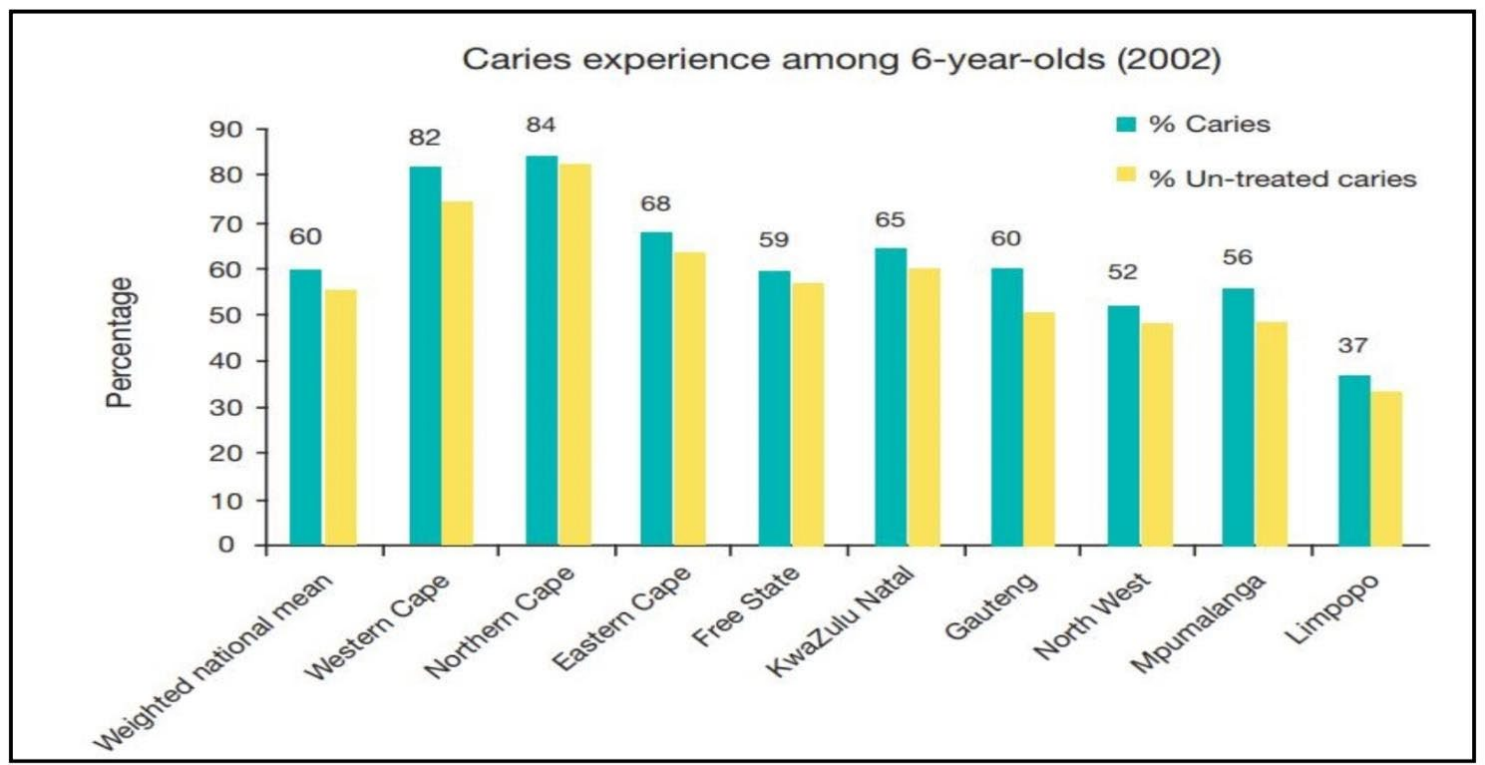
Prevalence of caries among children by province in South Africa, 2002. Source: Smit, et al. [18]

In South Africa, the first and only national oral health survey among children was conducted in 2002. The survey showed a high (60%) prevalence of caries among children below age 15, especially among 6-year-olds (Fig. 1) [18, 19].

Further, cross-sectional studies show that the prevalence of dental caries in the permanent and primary dentition were 26% and 30% among grade 1 and secondary school learners respectively in the Gauteng Province in 2013 [20]. In KwaZulu-Natal Province, 73% of 6-year-olds had dental caries in 2013 [21], whereas the prevalence of caries among children was over 70% in the Western Cape Province, [22, 23] with an unmet treatment need of 94% [23]. In addition the effects of dental caries in children has been shown to increase school absenteeism, affecting children’s educational performance [24, 25].

The above effects are possibly a reflection of the large quantities of dietary sugar consumed in South Africa. Between 2015 and 2020, consumption of sugary drinks alone (measured by off-trade sales) increased from 6,520 million liters to 8,182 million liters, representing a 25.5% increase during the period [26]. Before this, South Africans had already increased their sugar intake. For instance, among adults, added sugar consumption per capita for rural men increased from 28 g/d in 2005 to 68 g/day in 2010, while that of urban men increased from 45 g/d to 74 g/d for the same period. During 2005–2010, added sugar intake rose from 27 g/d to 66 g/d for rural women, while urban women increased their intake from 47 g/d to 79 g/d [27]. For pre-teens, added sugar intake ranged between 50 g/d and 100 g/day for teenagers [4, 28].

It is recommended that added sugar intake be restricted to 24 g/d for adult women and 36 g/d for adult men. For children in the ages of 2–18, added sugar intake should be limited to 24 g/d or lower, while sugary beverages be limited to 8 ounces or less (240 mL) a week [29]. Children, however, consume more sugar than recommended due to the industry marketing strategies that target children. A recent analysis of advertising data showed that the soft drinks industry targets children who are known to request beverage or food items they see advertised [30]. It is therefore not surprising that dental caries has become a major public health problem.

South Africa’s private and public health sectors provide oral health services to the population. However, due to the high level of income poverty and inequality coupled with the expensive nature of services in the private sector, 85% of the population relies on the public sector for their healthcare needs [31]. The public health sector with poor infrastructure and inadequate personnel therefore finds itself unable to address the disease burden and hence many children remain untreated for caries [18]. The learners are limited from accessing preventive and curative oral healthcare, starting from the least expensive preventative procedures such as Acidulated Phosphate Fluoride (APF) application, atraumatic restorative treatment (ART), and fissure sealants [32–35] to costly curative procedures such as extractions, restorations and root canal treatments. Achieving reduction in sugar consumption has also become a challenge due to industry push for higher sales volume for foods and beverages.

Except for the sugar reduction strategies, the above existing preventative regiments provide a vehicle for the uptake of fluoride among learners in the prevention of dental caries. The success of fluoride in the control and prevention of dental caries has been well documented [38–40]. For instance, community water fluoridation in the US showed substantial benefits to children [40]. In South Africa, and many LMICs, however, fluoride affordability continues to be a challenge and controlled water fluoridation has not yet been implemented [38, 41]. This implies that the majority of school learners may not have access to fluoride either through toothpaste or through water. In addition to fluoride usage, services like ART and fissure sealants have been shown to provide additional preventative benefits [34, 42]. These two interventions utilize glass-Ionomer or bioactive glass in order to repel bacterial plaque and re-mineralize tooth surfaces for the prevention of dental caries [35, 42]. As sugar consumption is a major dietary contributor to the cause of dental caries, the use of sugar reduction approach in policy or dietary regulation has shown to be effective in reducing the caries incidence [12, 13, 43]. Public health resources should therefore be prioritized to the prevention of dental decay among children and adolescents with evidenced-based approaches [43].

The National Department of Health’s policy aspiration is to improve oral health access, prevention and care and has put efforts into school-based oral health programs. Oral health personnel go into schools to provide oral health screenings, education and preventative care as mentioned above [44, 45]. The benefits of such programs are to promote the adoption of lifestyles that promote good health, and enable learners and staff to take action for a healthier community and widen access to care [46]. In addition, preventing dental caries among children is key in achieving better oral health during adulthood since caries in childhood has a lingering effect and is a predictor for adult caries and other oral conditions [2].

In South Africa, however, the limited studies on economic analysis of dental caries prevention programs have been short of providing information on the preventive health benefits from such oral health programs [47–49]. Hence few cost-effectiveness analysis on caries prevention exist for South Africa [22, 50]. Due to limited literature, this study sought to address the evidence gap by conducting an economic analysis of the costs and health benefits of the various dental caries preventative strategies offered to learners in South African public schools. Such an analysis is important for policy making and priority setting for oral health service delivery.

The key objectives of the study were to estimate the lifetime cost of adopting each intervention, the health benefits associated with each preventive strategy and to determine which of the strategies is most cost-effective. To our knowledge, this is the first study to use Markov modelling techniques to analyze the cost-effectiveness of five caries prevention strategies for children in South Africa.

## Methods

### Study design

**Fig. 2:**
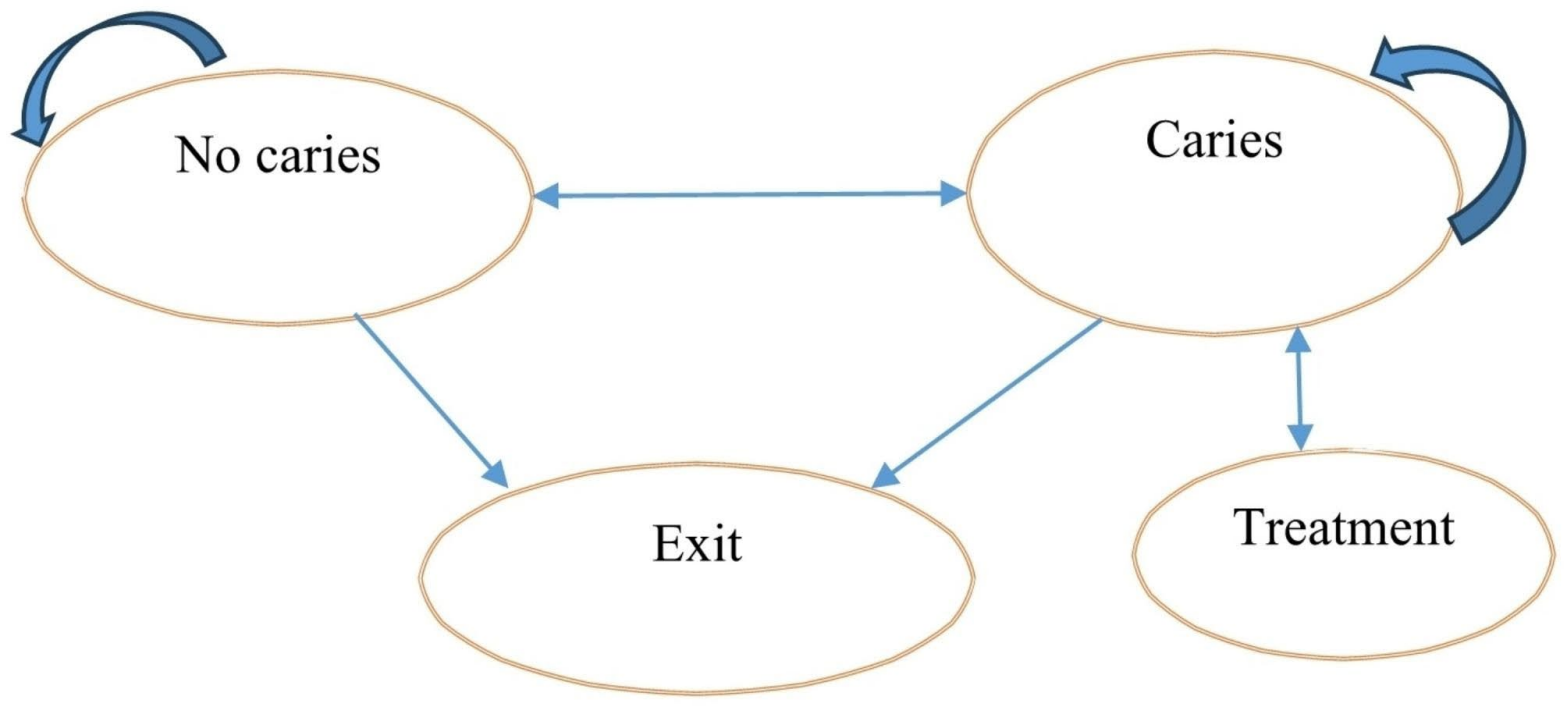
Markov Model for oral health

A Markov model was used to evaluate the cost-effectiveness of dental caries preventative strategies in children. We simulated the interventions for dental caries among a hypothetical learner population of 10,000 who start school at age 5 and exit once they have reached age 15. In Fig. 2, the model assumes that there are four states in which a learner could find him/herself. The learner can move from “no caries state” to “caries state” and then treatment state or even exit the group either due to school dropout, relocation, or change from public school to private school. The model was adapted from previous studies on the cost-effectiveness of oral health interventions [36, 51].

Given that majority of South Africans receive health care from the public sector, the study perspective is that of a public sector healthcare payer. Our chosen time horizon is 10 years, informed by the fact that learners start school at age 5 (Grade 1 applicants must be at least 5 years old) [52] and complete primary school at age 15. A discount rate of 5% was used based on national guidelines [53]. The model was designed in Microsoft Excel, with the aid of Visual Basic for Applications (VBA)^1^.

### Prevention strategies

Fissure sealants, fluoride applications, atraumatic restorative treatment (ART) and reduction in added sugar intake among children (which may be achieved through, for example, taxation and marketing restrictions) were our main strategies. In this study, we model the caries-effect of 30 g/d reduction in added sugar intake to achieve the recommended 24 g/d or less consumption. In the case of other programs, we model the caries-effect of annual implementation of caries prevention programs.

### Oral health outcomes

Since our start age is age 5, we use dmft (decayed, missing, and filled primary teeth) and DMFT (decayed, missing, and filled permanent teeth) scores as the outcome for each intervention [4, 33, 36]. The dmft/DMFT is used as a marker to capture the caries experience among children. Thus, the percentage reduction in dmft/DMFT resulting from each program was calculated. The comparator “do nothing” had no averted dmft/DMFT. The dmft score is used among children under 6 years who mostly have primary teeth, while DMFT score is used for children 6 years or older who have permanent teeth. However, for the purposes of this study, we use DMFT to capture caries experience among children under 6 (dmft) and 6 years or older (DMFT).

### Costs

Direct costs associated with each prevention strategy were obtained from the literature (Table 1). These costs included expenditures on items such as personnel, equipment, materials and supplies as well as the cost of implementing sugar reduction strategies such as food taxes and marketing restrictions [47, 55]. All costs were in South African Rand (ZAR) and adjusted and presented in 2022 constant prices using the consumer price index.

### Sources of data

The study used data from published and grey literature and other publicly available information. The data for the parameters and sources were compiled in Microsoft Excel. Table 1 presents the parameters used in the model and their sources. Expert opinion was relied upon in the absence of publicly available data for other variables, whenever necessary (Table 2).

**Table 1:** Summary of model inputs and sources

Parameter	Distribution	Mean (SD)	Description	Source
Costs per learner (ZAR)				
Tooth brushing	Gamma	548.33 (544.29)		[22]
ART	Gamma	89.24	Cost of screening and /or delivering ART, APF-Gel, fissure sealant and tooth brushing to a learner.	[47]
APF-Gel	Gamma	193.73		[47]
Fissure sealant	Gamma	59.71		[47]
Screening	Gamma	525.61		[47]
Sugar reduction ^#^	Gamma	1.25		[55]
**Outcomes/Effectiveness**				
DMFT, %				
			Percentage reduction of DMFT resulting from the intervention	
APF-Gel	Beta	21 (4)		[33]
Fissure sealant	Beta	50 (8)		[33]
ART	Beta	72 (25)		[56]
Tooth brushing	Beta	60 (47)		[57]
Sugar reduction	Beta	31.89 (0.18)		[58]
**Probabilities**				
Mean DMFT at baseline*		Inserted as table	DMFT expressing caries experience	[4]
Prob_caries**		Inserted as table	Probability of experiencing caries (time dependent)	[19]
Discount rate		0.05	Discount rate to account for the time value of money	[53]
Prob_exit**		Inserted as table	Probability of exiting sample (time dependent)	[59]
Prob_recurrence		0.188	probability of caries recurrence	[60]
Prob_untreated**		0.7	Probability of untreated caries	[61]

** Age dependent variables, see Table 3 in appendix. Due to lack of data on standard deviation for some variables, we used 10% of the mean to represent the standard deviation [62]. ^#^ Cost of sugar reduction program includes cost of implementing SSB taxation and marketing restrictions. Note that the costs are inflation-adjusted. Prob_caries: probability of developing caries; Prob_exit: probability of a child leaving the sample, based on school drop-out rates

### Data analysis

To measure the cost-effectiveness of prevention programs, a Markov model was used. A Markov modelling is a health economics tool used to depict the movement of a hypothetical patient cohort between different health states over time.

### Assumptions of the model

Except for sugar reduction, all interventions incorporate an oral health screening. We assumed that following screening, learners with caries and other oral diseases will be treated on site or referred to a health facility, if needed. We also assumed the following.

1. DMFT increases annually by 0.59% through the follow-up period.
2. All interventions are mutually exclusive, meaning that two or more interventions cannot be implemented simultaneously.
3. Linear relationship between added sugar consumption and dental caries, every 1 g/d leads to 0.0128 increase in DMFT, and we model a reduction in current sugar consumption by 30 g/d.

### Cost-effectiveness analysis

To calculate the ICER, each intervention was compared to the “do nothing” option, and the incremental cost was divided by the incremental effectiveness. We then ranked all interventions in ascending order based on effectiveness (averted DMFT) and compared each intervention to the next most effective intervention keeping in mind the principle of dominance. Using cost-effectiveness (willingness-to-pay (WTP)) threshold of ZAR38,500, [63] we calculated the net monetary benefit (NMB) by multiplying the cost-effectiveness or the WTP threshold by the health gain and subtracted the total cost of the intervention.

### Sensitivity analysis

Uncertainty in model parameters was characterized through Probability Sensitivity Analysis (PSA). Cost and effectiveness parameters were varied using gamma and beta distributions, respectively (Table 1). Cost-effectiveness acceptability curve (CEAC) was produced for each intervention to establish the probability that a given intervention is cost-effective. The CEAC, which is derived from the joint distribution of the effects and costs of an intervention summarizes the uncertainty in estimates of cost-effectiveness. The PSA used 1000 Monte Carlo Simulations.

## Results

### Deterministic model

We simulated the cost and effectiveness of oral health intervention programs for a hypothetical learner population of 10,000. The cohort started at age 5 and exited the program after attaining age 15 or due to other reasons. Screening was included in all (except sugar reduction) programs and its cost of implementation was ZAR43.78 million for the 10 years for 10,000 learners. Table 2 presents the results from the deterministic model, which indicates that the total lifetime cost of implementing a sugar reduction program was ZAR91,380. This cost was incurred as a result of implementing measures such as taxation and marketing restrictions that reduce sugar intake.

**Table 2:** Cost-effectiveness of oral health interventions for children

Intervention	Total Costs	DMFT**	Average lifetime cost	ICER (compared to Do Nothing)
Do nothing			Ref	Ref
APF-Gel	54,024,439	42,010	5,402	1,286
Sugar reduction	91,380	63,762	9	1.43
Tooth brushing	72,779,917	74,018	7,278	983
Fissure sealant	44,633,687	100,024	4,463	446
ART	45,057,166	144,035	4,506	313

ICER: incremental cost-effectiveness ratio

** Number of teeth averted for DMFT

All costs are in ZAR

The total cost for implementing (inclusive of screening cost of ZAR43.78 million) was ZAR54 million for APF-Gel program, and ZAR45 million for ART program (Table 2). The APF-Gel program averted 42,010 DMFT (the least) of all interventions, whereas ART program averted 144,035 (the highest). There was no financial cost or averted DMFT associated with the “do nothing” option. The average lifetime cost per learner ranged from ZAR9 for sugar-reduction program to ZAR7278 for tooth brushing program during the 10-year period.

The results shown in Table 3 indicate that APF-Gel program had the highest ICER, ZAR1,286 per DMFT averted compared to the “do nothing” option.

**Table 3:** ICER and Net Monetary Benefit

Intervention	ICER based on ranking	NMB
Do nothing		
APF-Gel	1286	1,563,370,809
Sugar reduction	-2480	2,454,739,193
Tooth brushing	7087	2,776,916,473
Fissure sealant	-1,082	3,806,307,380
ART	10	5,500,297,970

Compared to the tooth brushing program, the negative ICER of ZAR1082 indicate that fissure sealant program resulted in cost savings, indicating that fissure sealant program was more cost-effective than tooth brushing program. Similarly, comparing sugar-reduction strategy to APF-Gel shows that sugar-reduction resulted in cost saving per DMFT averted at ZAR2480. The results from the net monetary benefit show that ART provides the highest benefits, while APF-Gel produced the lowest benefits.

### Probabilistic sensitivity analysis (PSA)

**Fig. 3:**
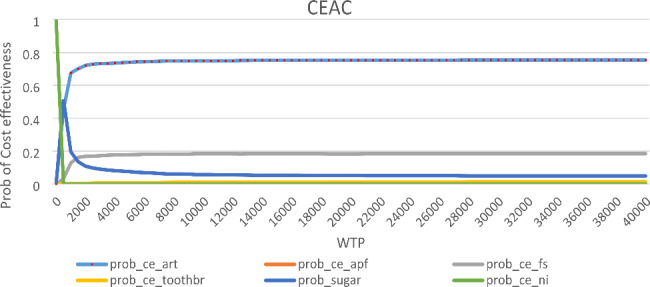
Cost-effectiveness acceptability curve (CEAC)

**Fig. 4:**
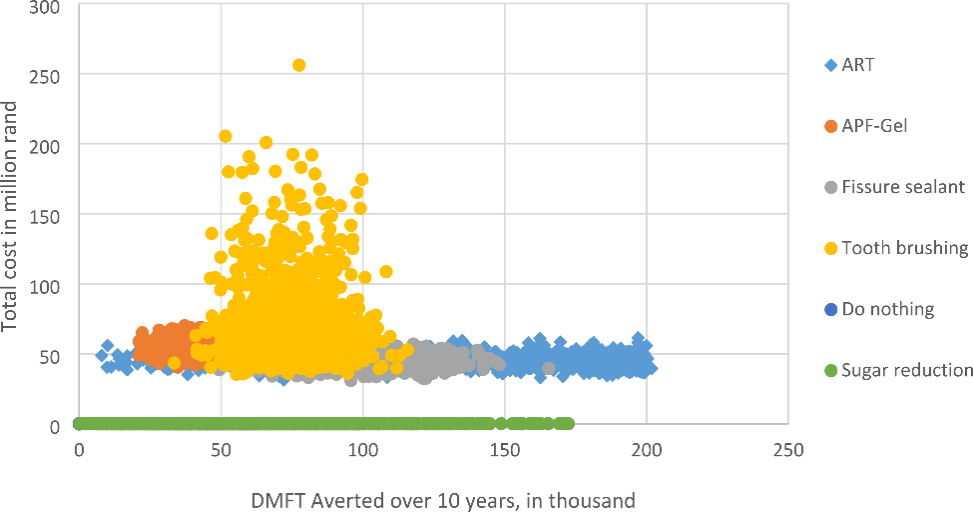
Distribution of cost and effectiveness for oral health interventions

To provide a credible range for the estimates, we conducted probability sensitivity analysis. Table 4 presents the summarized results from the 1000 Monte Carlo Simulations. The results were similar to those obtained under the deterministic model. The cost-effectiveness acceptability curves (CEACs) and the distribution (a scatter plot) of costs and effectiveness are shown in Figs. 3 and 4, respectively. The CEACs shown in Fig. 3 indicate the probability that an intervention is cost-effective when compared with the alternative, given the data, for a range of WTP thresholds.

**Table 4:** Cost-effectiveness results based on ranking

Intervention	Costs	DMFT**	Average lifetime cost (ZAR)	ICER (compared to Do Nothing)	NMB	SD, Costs	SD, DMFT
Do nothing							
APF-Gel	54,091,579	41,706	5,409	1,297	1,551,572,826	281,589	510
Sugar reduction	91,392	62,964	9	1.45	2,424,034,402	552	2,194
Tooth brushing	73,078,877	74,527	7,308	981	2,796,225,000	1,802,852	807
Fissure sealant	44,705,881	100,410	4,471	445	3,821,083,511	274,813	988
ART	45,134,249	141,174	4,513	320	5,390,046,733	274,207	3,072

ICER: incremental cost-effectiveness ratio

** Number of teeth averted for DMFT

SD: standard deviation

All costs are in ZAR

Figure 3 shows that ART program has 0.75 probability of being cost-effective at almost all cost-effectiveness thresholds, while fissure sealant program had 0.2 probability of being cost-effective. Supervised tooth brushing and “do nothing” options had zero probability of being effective, except at WTP of ZAR0 whereas “do nothing” had a 100% chance of being cost-effective. Also, ART appeared to produce higher oral health benefits at a lower cost (Fig. 4).

## Discussion

Building on previous economic analysis on dental caries in South Africa, [22, 47, 50] this study analyzed the cost-effectiveness of five caries preventative programs for schoolchildren in South Africa. The comparator was “do nothing” which was associated with no financial cost for government. The results show that all caries preventative programs were cost-effective compared to the status-quo, “doing nothing”, at a willingness-to-pay threshold greater than zero per DMFT averted. The probability of having a cost-effective program was about 75% for the ART intervention, at almost all WTP thresholds. In the case of sugar reduction, the CEAC showed a probability of cost-effectiveness of 0.5 at a WTP threshold of ZAR500. WTP thresholds higher than ZAR500 recorded lower probability of cost-effectiveness for sugar reduction. Both ART and sugar reduction programs had about 50% chance of being cost-effective at WTP of ZAR500. At this WTP, the NMB for APF-Gel and supervised tooth brushing programs were negative, while sugar reduction (ZAR33 million), ART (ZAR27 million) and fissure sealant (ZAR5.4 million) were positive. The Monte Carlo simulations showed that the cost-effectiveness of the interventions compared to the status-quo was not sensitive to parameter variation. The results showed that doing nothing about dental caries among children was not cost-effective compared to the alternatives at a WTP above zero.

Based on the net monetary benefit estimated at WTP of ZAR38500, the ART program was the most cost-effective, producing about ZAR5.5 billion worth of oral health benefits. This was followed by a fissure sealant program with ZAR3.8 billion worth of benefits. The least cost-effective program among the five alternative interventions was APF-Gel program, which recorded the highest incremental cost (ZAR1825) per averted DMFT compared to the baseline intervention, “do nothing” and had the lowest NMB, ZAR1.6 billion. The findings are consistent with previous studies which have shown that ART [64] and fissure sealants [65] are cost-effective interventions in caries prevention. Findings from Chile show that the use of APF-Gel in caries prevention was less cost-effective compared to other interventions such as supervised tooth brushing programs [33]. Studies have also found that a reduction in added sugar intake, using tools like taxation [62, 66] and front of pack labelling [67], can lower dental caries incidence significantly among both children and adults and both are therefore regarded as cost-effective relative to “do nothing”. Prior studies were impact assessment studies that did not compare the health outcomes and cost of sugar reduction from SSB taxation to other caries prevention programs. When compared to “do nothing, reducing added sugar intake by 30 g/d averted approx. 63000 DMFT over 10 years among children. When compared to the other alternatives such as tooth brushing, fissure sealant and ART, however, sugar reduction alone was not the most cost-effective. Indeed, in Thailand,it has been shown that tax-based sugar reduction strategy alone may not yield expected oral health benefits due to substitution effects which justifies inclusion of, if possible, all added sugar sources for tax [68].

The high cost-effectiveness of ART may stem from the fact that the ART procedure relies on less expensive portable hand instruments and can be performed in any location. The ART procedure which removes carious tissues and fills cleaned cavities and adjacent fissures with a high-viscosity glass ionomer cement has shown high survival rates for restorations in both primary and permanent teeth [34, 42]. In South Africa, the ART procedures have been used among dental practitioners since its introduction in the country in 1996 [69]. This is mainly due to its economical and restorative advantages. A clinical evaluation of ART in some schools in Johannesburg showed that 98% of the ART restorations were caries-free after a year [32], and has been shown to cost 50% lower than conventional restorative procedures [49].

In a recent cost-effectiveness analysis between fluoride varnish and tooth brushing programs in Cape Town, [22] results showed that the application of fluoride varnish, which is similar to APF-Gel, did not provide any substantial caries-preventive benefits. This is consistent with our findings regarding APF-Gel in our study. Although daily supervised tooth brushing program with fluoridated toothpaste in school is an effective caries preventitive procedure, [17] we found that ART, fissure sealants and sugar reduction provided substantial gains in oral health improvement at relatively lower cost. This assists in prioritizing oral health preventative interventions in school settings. However one needs to take into consideration that these approaches need not be used in isolation, as resources permit, they can be used in various combinations in order to maximize dental caries prevention at school settings [70]. The effectiveness of caries preventative programs has been suggested to be greater when a combination of these preventive measures is used [33].

### Limitations

The study assesses the cost and benefits from a public healthcare payer perspective. Therefore, non-healthcare costs associated with oral health diseases such as the loss of school days and associated poor academic performance were not estimated. Nor was the cost associated with a parent taking off time to take the child to the clinic. Similarly, healthcare costs borne by the private sector were not considered. Another limitation relates to the lack of comprehensive data on the effectiveness and quality of implementing the strategies in the context of South Africa. Nonetheless, this study assumed that each intervention was implemented separately which may underestimate the benefits of the programs. Further, this study modelled sugar reduction of 30 g/d, suggesting that higher reductions may result in larger oral health benefits, especially children consuming 100 g/d.

## Conclusion

There is a high prevalence of dental caries among children in South Africa and most children go untreated. We have analyzed the cost-effectiveness of government strategies to improve oral health among learners in South Africa using a simple Markov model for 10,000 learners in the public sector. We found that all school-based caries prevention programs were cost-effective compared to the status quo of doing nothing. However, among the five other alternative programs, ART was found to be the most cost-effective intervention, whilst APF-Gel was the least cost-effective intervention, based on the net monetary benefit and the cost-effectiveness acceptability curve. The findings provide support for the use of ART procedures in caries preventative programs among learners in South Africa. Given that sugar consumption is a major cause of caries and tooth decay among children, caries preventative programs such as the ART and fissure sealant programs should be accompanied by sugar reduction at the population level.

### Electronic supplementary material

Below is the link to the electronic supplementary material.

Supplementary Material 1

## Data Availability

The study uses publicly available data for analysis as given in the references provided (see Table 1). However, the authors will make the data available on reasonable request.

## Footnotes

1 Kibohut [54] provides valuable learning resources for readers interested in building spreadsheet-based Markov models.

## Declarations

### Patient involvement

No patients were involved.

## References

[1] Bernabe E, Marcenes W, GBD Oral Disorders Collaborators (2020). Global, regional, and national levels and trends in burden of oral conditions from 1990 to 2017: a systematic analysis for the global burden of disease 2017 study. J Dent Res.

[2] Bernabé E, Sheiham A (2014). Age, period and cohort trends in caries of permanent teeth in four developed countries. Am J Public Health.

[3] Chrisopoulos S, Harford J, Ellershaw A. Oral health and dental care in Australia: key facts and Fig. 2015 ed. Australian Institute of Health and Welfare; 2016.

[4] Steyn NP, Temple NJ (2012). Evidence to support a food-based dietary guideline on sugar consumption in South Africa. BMC Public Health.

[5] Chi DL, Hopkins S, O’Brien D (2015). Association between added sugar intake and dental caries in yup’ik children using a novel hair biomarker. BMC Oral Health.

[6] Cheng Y-C, Huang H-K, Wu C-H (2014). Correlation between dental caries and diet, oral hygiene habits, and other indicators among elementary school students in Xiulin Township, Hualien County, Taiwan. Tzu Chi Medical Journal.

[7] Sheiham A, James WPT (2014). A reappraisal of the quantitative relationship between sugar intake and dental caries: the need for new criteria for developing goals for sugar intake. BMC Public Health.

[8] Karjalainen S, Tolvanen M, Pienihäkkinen K (2015). High sucrose intake at 3 years of age is associated with increased salivary counts of mutans streptococci and lactobacilli, and with increased caries rate from 3 to 16 years of age. Caries Res.

[9] van Loveren C (2019). Sugar restriction for caries prevention: amount and frequency. Which is more important?. Caries Res.

[10] Ruottinen S, Karjalainen S, Pienihäkkinen K (2004). Sucrose intake since infancy and dental health in 10-year-old children. Caries Res.

[11] Kumar D, Gandhi K, Maywad S (2020). Prevalence and correlation of Dental Caries with its specific risk factors in 5–15-year-old School-going children in Urban Population of Ghaziabad. Int J Clin Pediatr Dentistry.

[12] Bernabé E, Vehkalahti MM, Sheiham A (2014). Sugar-sweetened beverages and dental caries in adults: a 4-year prospective study. J Dent Res.

[13] Bernabé E, Ballantyne H, Longbottom C (2020). Early introduction of sugar-sweetened beverages and caries trajectories from age 12 to 48 months. J Dent Res.

[14] Meier T, Deumelandt P, Christen O (2017). Global burden of sugar-related dental diseases in 168 countries and corresponding health care costs. Journal of dental research. J Dent Res.

[15] Kleinberg I (2002). A mixed-bacteria ecological approach to understanding the role of the oral bacteria in dental caries causation: an alternative to Streptococcus mutans and the specific-plaque hypothesis. Crit Reviews Oral Biology Med.

[16] Dörfer C, Benz C, Aida J (2017). The relationship of oral health with general health and NCDs: a brief review. Int Dent J.

[17] Samuel SR, Acharya S, Rao JC (2020). School Interventions–based Prevention of Early-Childhood Caries among 3–5‐year‐old children from very low socioeconomic status: two‐year randomized trial. J Public Health Dent.

[18] Smit D, Barrie R, Louw A (2017). The burden of dental caries in the western cape and a recommended turn-around strategy. South Afr Dent J.

[19] Singh S (2011). Dental caries rates in South Africa: implications for oral health planning. South Afr J Epidemiol Infect.

[20] Molete M, Igumbor J, Stewart A (2019). Dental status of children receiving school oral health services in tshwane. South Afr Dent J.

[21] Reddy M, Singh S (2015). Dental caries status in six-year-old children at health promoting schools in KwaZulu-Natal, South Africa. South Afr Dent J.

[22] Effenberger S, Greenwall L, Cebula M, et al. Cost-effectiveness and efficacy of fluoride varnish for caries prevention in south african children: a cluster‐randomized controlled community trial. Community Dentistry and Oral Epidemiology; 2021. 10.1111/cdoe.12702 34676577

[23] Mohamed N, Barnes JM. Early childhood caries and dental treatment need in low socio-economic communities in Cape Town, South Africa. Health SA Gesondheid 2018;23. 10.4102/hsag.v23i0.1039 PMC6917374 31934368

[24] Jackson SL, Vann WF, Kotch JB (2011). Impact of poor oral health on children’s school attendance and performance. Am J Public Health.

[25] Krisdapong S, Prasertsom P, Rattanarangsima K (2013). School absence due to toothache associated with sociodemographic factors, dental caries status, and oral health-related quality of life in 12‐and 15‐year‐old T hai children. J Public Health Dent.

[26] Eurominitor International. Soft drinks in South Africa. Eurominitor International; 2021.

[27] Vorster HH, Kruger A, Wentzel-Viljoen E (2014). Added sugar intake in South Africa: findings from the adult prospective urban and rural epidemiology cohort study. Am J Clin Nutr.

[28] Temple NJ, Steyn NP (2013). Sugar and health: a food-based dietary guideline for South Africa. South Afr J Clin Nutr.

[29] Health HSoP. Added Sugar n.d [Available from: https://www.hsph.harvard.edu/nutritionsource/carbohydrates/added-sugar-in-the-diet/#:~:text=The%20AHA%20suggests%20a%20stricter,of%20sugar)%20for%20most%20men. accessed 01 Jan 2023.

[30] Boachie MK, Goldstein S, Kruger P (2023). Beverage industry’s advertising and airtimes in South Africa from 2013 to 2019 target children and families. J Public Health Res.

[31] Statistics South Africa (Stats SA). General Household Survey, 2019. Pretoria, 2020.

[32] Mickenautsch S, Kopsala J, Rudolph M (2000). Clinical evaluation of the ART approach and materials in peri-urban farm schools of the Johannesburg area. SADJ: J South Afr Dent Association.

[33] Mariño R, Fajardo J, Morgan M (2012). Cost-effectiveness models for dental caries prevention programmes among chilean schoolchildren. Community Dent Health.

[34] Molina GF, Cabral RJ, Frencken JE (2009). The ART approach: clinical aspects reviewed. J Appl Oral Sci.

[35] Nicholson JW (2022). Periodontal Therapy using Bioactive Glasses. Rev Prosthes.

[36] Nguyen TM, Tonmukayakul U, Warren E (2020). A Markov cost-effective analysis of biannual fluoride varnish for preventing dental caries in permanent teeth over a 70‐year time horizon. Health Promotion Journal of Australia.

[37] Ormsby RT, Hosaka K, Evdokiou A (2022). The Effects of vitamin E analogues α-Tocopherol and γ-Tocotrienol on the human osteocyte response to Ultra-high Molecular Weight Polyethylene wear particles. Prosthesis.

[38] Gkekas A, Varenne B, Stauf N (2022). Affordability of essential medicines: the case of fluoride toothpaste in 78 countries. PLoS ONE.

[39] Petersen PE, Ogawa H (2016). Prevention of dental caries through the use of fluoride – the WHO approach. Community Dent Health.

[40] Slade G, Grider W, Maas W (2018). Water Fluoridation and Dental Caries in U.S. children and adolescents. J Dent Res.

[41] Kroon J, Wyk, PJv (2012). A model to determine the economic viability of water fluoridation. J Public Health Dent.

[42] Amorim Rd, Frencken J, Raggio D (2018). Survival percentages of atraumatic restorative treatment (ART) restorations and sealants in posterior teeth: an updated systematic review and meta-analysis. Clin Oral Invest.

[43] Horst JA, Tanzer JM, Milgrom PM (2018). Fluorides and other preventive strategies for tooth decay. Dental Clin N Am.

[44] Reddy M, Singh S (2015). Viability in delivering oral health promotion activities within the Health promoting Schools Initiative in KwaZulu-Natal. South Afr J Child Health.

[45] Mpho Molete AS, Jude Igumbor (2020). Implementation fidelity of school oral health programs at a District in South Africa. PLoS ONE.

[46] Rebecca Langford C, Bonell H, Jones, et al. Obesity prevention and the Health promoting schools framework: essential components and barriers to success. Int J Behav Nutr Phys Activity. 2015;12(15). 10.1186/s12966-015-0167-7. 10.1186/s12966-015-0167-7 PMC4330926 25885800

[47] Molete M, Chola L, Hofman K (2016). Costs of a school-based dental mobile service in South Africa. BMC Health Serv Res.

[48] Mahomed O, Mthethwa J (2022). Estimating the cost of oral health services for 2018/19 financial year at public health facilities in two KwaZulu-Natal districts, South Africa: a retrospective study. J Int Oral Health.

[49] Mickenautsch S, Munshi I, Grossman E (2002). Comparative cost of ART and conventional treatment within a dental school clinic. SADJ: J South Afr Dent Association = Tydskrif van die Suid-afrikaanse Tandheelkundige Vereniging.

[50] Kroon J, Van Wyk PJ (2012). A retrospective view on the viability of water fluoridation in South Africa to prevent dental caries. Commun Dent Oral Epidemiol.

[51] Warren E, Pollicino C, Curtis B (2010). Modeling the long-term cost-effectiveness of the caries management system in an australian population. Value in Health.

[52] Government of South Africa. Admission to school: Government of South Africa; n.d. [Available from: https://www.gov.za/services/basic-education/admission-school accessed 21 August 2023.

[53] National Department of Health. The guidelines for pharmacoeconomic evaluations of medicines and scheduled substances. Pretoria, 2010.

[54] Kibohut. Markov Models in Excel. : Kibohut; [Available from: http://kibohut.com/download/index.php accessed Jan 15 2021.

[55] Cecchini M, Sassi F, Lauer JA (2010). Tackling of unhealthy diets, physical inactivity, and obesity: health effects and cost-effectiveness. The Lancet.

[56] Gibilini C, de Paula JS, Marques R (2012). Atraumatic restorative treatment used for caries control at public schools in Piracicaba, SP, Brazil. Brazilian J Oral Sci.

[57] Van der Walt M, Van Wyk PJ, Bester J (2018). The effectiveness of a tooth brushing programme for children in the Ehlanzeni district of Mpumalanga. South Afr Dent J.

[58] Rugg-Gunn AJ, Hackett AF, Appleton DR (1984). Relationship between dietary habits and caries increment assessed over two years in 405 english adolescent school children. Arch Oral Biol.

[59] Staff Writer. This is the school drop out rate in South Africa. BusinessTech; 2020.

[60] Jiang H, Shen L, Qin D (2019). Effects of dental general anaesthesia treatment on early childhood caries: a prospective cohort study in China. BMJ open.

[61] Van Wyk C, Van Wyk PJ (2010). Trends in dental caries prevalence, severity and unmet treatment need levels in South Africa between 1983 and 2002: scientific. South Afr Dent J.

[62] Jevdjevic M, Trescher A-L, Rovers M (2019). The caries-related cost and effects of a tax on sugar-sweetened beverages. Public Health.

[63] Edoka IP, Stacey NK (2020). Estimating a cost-effectiveness threshold for health care decision-making in South Africa. Health Policy Plann.

[64] Tonmukayakul U, Forrest H, Arrow P (2021). Cost-effectiveness analysis of atraumatic restorative treatment to manage early childhood caries: microsimulation modelling. Aust Dent J.

[65] Griffin S, Naavaal S, Scherrer C (2016). School-based dental sealant programs prevent cavities and are cost-effective. Health Aff.

[66] Schwendicke F, Thomson WM, Broadbent JM (2016). Effects of taxing Sugar-Sweetened Beverages on Caries and Treatment costs. J Dent Res.

[67] Jevdjevic M, Wijn SRW, Trescher A-L et al. Front-of-Package Food labeling to reduce caries: economic evaluation. J Dent Res 2021;100(5). 10.1177/0022034520979147 PMC8058828 33331232

[68] Nipaporn Urwannachotima P, Hanvoravongchai JP, Ansah (2020). Impact of sugar-sweetened beverage tax on dental caries: a simulation analysis. BMC Oral Health.

[69] Mickenautsch S, Frencken JE (2009). Utilization of the ART approach in a group of public oral health operators in South Africa: a 5-year longitudinal study. BMC Oral Health.

[70] Mishu MRFMP, Jahangir F (2022). The effectiveness of behaviour change interventions delivered by non-dental health workers in promoting children’s oral health: a systematic review and meta-analysis. PLoS ONE.

