# Supplementary material for: Cost-effectiveness of dental caries prevention strategies in South African schools
Source: BMC Oral Health. 2023 Oct 28;23:814. doi: 10.1186/s12903-023-03474-1 (PMC10613394; doi:10.1186/s12903-023-03474-1)
Supplement: Supplementary file 1 — Supplementary Material 1 [file 12903_2023_3474_MOESM1_ESM.docx]

**Appendix**

**Table 1: Baseline Characteristics**

| Age | Prob_caries | Prob_exit | DMFT_baseline |
| --- | --- | --- | --- |
| 5 | 0.506 | 0.002 | 2.40 |
| 6 | 0.603 | 0.003 | 2.90 |
| 7 | 0.603 | 0.003 | 2.90 |
| 8 | 0.603 | 0.006 | 2.90 |
| 9 | 0.603 | 0.013 | 2.90 |
| 10 | 0.603 | 0.027 | 2.90 |
| 11 | 0.603 | 0.044 | 2.90 |
| 12 | 0.369 | 0.105 | 1.10 |
| 13 | 0.369 | 0.148 | 1.10 |
| 14 | 0.510 | 0.241 | 1.10 |
| 15 | 0.510 | 0.173 | 1.90 |
